# Comparison of pupillary diameter variation between conventional phacoemulsification versus femtosecond laser-assisted cataract surgery

**DOI:** 10.5935/0004-2749.20210091

**Published:** 2022

**Authors:** Caroline Schiave Germano, Renato Antunes Schiave Germano, Felipe Biscegli Cid, Flavio Augusto Schiave Germano, Pedro Carlos Carricondo, Jorge Estefano Germano

**Affiliations:** Department of Ophthalmology, Santa Casa de Misericórdia de São Paulo, São Paulo, SP, Brazil.; Department of Ophthalmology, Santa Casa de Misericórdia de São Paulo, São Paulo, SP, Brazil. Department of Ophthalmology, Universidade de São Paulo, São Paulo, SP, Brazil; Centro de Exclência em Oftalmologia, Bauru, SP, Brazil

**Keywords:** Cataract, Miosis, Phacoemulsification, Laser, Pupil, Catarata, Miose, Facoemulsificação, Laser, Pupila

## Abstract

**Purpose:**

To evaluate and compare variations in pupillary diameter before and after cataract surgery by conventional phacoemulsification versus femtosecond laser assisted cataract surgery, using LDV Z8 (Ziemer Ophthalmic). We also evaluated the relationship between pupillary diameter and surgery time and ultrasound time.

**Methods:**

Prospective comparative study, carried out at the Center of Excellence in Ophthalmology, Brazil. We included 79 eyes of 67 patients with nuclear opacity. The patients were divided into the control and study groups, including those who underwent cataract surgery with manual phacoemulsification, and femtosecond laser assisted cataract surgery, respectively. All surgeries were performed by the same experienced surgeon. All patients received topical non-steroidal anti-inflammatory drugs on the day before surgery and the same mydriatic eye drops preoperatively. To quantify pupillary size, measurements were performed using a surgical compass: anterior to the phacoemulsification procedure and at the end of the surgery. In the study group, measurements after laser were added. Surgical time and phacoemulsification time were also analyzed.

**Results:**

No significant difference was found between the pre-femto × pre-phaco pupil size (8.69 ± 0.44 mm × 8.63 ± 0.72 mm; *p*=0.643), and the pupil size at the end of surgery (7.96 ± 0.98 mm × 7.78 ± 0.95 mm; *p*=0.480) and the mean time of surgery (*p*=0.780). However, in the femtosecond laser assisted cataract surgery group, a transient increase in pupillary diameter after laser treatment was observed, indicating a tendency for greater variation in the femto group.

**Conclusions:**

Although pupil size diameter was similar at the end of surgery, the femtosecond laser assisted cataract surgery group presented higher intraoperative pupil variation. The surgeon should be aware of pupil size diameter before surgery for better and safer performance of femtosecond laser assisted cataract surgery.

## Abstract (pt)

**Objetivo:**

Avaliar e comparar a variação do diâmetro pupilar antes e após a cirurgia de catarata por facoemulsificação convencional versus cirurgia de catarata assistida por laser de femtossegundo, usando o LDV Z8 (Ziemer Ophtalmic). Também avaliamos a relação entre o diâmetro pupilar com o tempo da cirurgia e o tempo de ultrassom.

**Métodos:**

Estudo comparativo prospectivo, realizado no Centro de Excelência em Oftalmologia, Brasil. Foram incluídos 79 olhos de 67 pacientes com opacidade nuclear. Os mesmos foram divididos em Grupo Controle, que foi submetido a cirurgia de catarata com facoemulsificação manual, e Grupo Estudo, com catarata assistida por laser de femtossegundo. Todas as cirurgias foram realizadas pelo mesmo cirurgião experiente. Todos os pacientes receberam antiinflamatório não esteróide tópico no dia anterior à cirurgia e o mesmo colírio midriático no pré-operatório. Para quantificar o tamanho da pupila, as medidas foram realizadas usando um compasso cirúrgico: anterior ao procedimento de facoemulsificação e ao final da cirurgia. No grupo de estudo, medidas após o laser foram adicionadas. O tempo cirúrgico e o tempo de facoemulsificação também foram analisados.

**Resultados:**

Não foi encontrada diferença significativa entre o tamanho da pupila pré-femto x pré-faco (8,69 ± 0,44 mm x 8,63 ± 0,72 mm; p=0,643), bem como o tamanho da pupila no final da cirurgia (7,96 ± 0,98 mm x 7,78 ± 0,95 mm; p=0,480) e o tempo médio de cirurgia (p=0,780). No entanto, no grupo de catarata assistida por laser de femtossegundo, houve um aumento transitório do diâmetro pupilar após o laser, indicando uma tendência para maior variação no grupo femto.

**Conclusões:**

Embora o diâmetro pupilar fosse semelhante ao final da cirurgia, o grupo com catarata assistida por laser de femtossegundo apresentou maior variação intraoperatória da pupila. Portanto, para uma cirurgia de catarata assistida por laser de femtossegundo mais eficiente e segura, o cirurgião deve estar ciente do tamanho do diâmetro pupilar antes do procedimento.

## INTRODUCTION

Cataract, the main cause of reversible blindness globally, is effectively treatable with surgery using one of the most common ophthalmic procedures^(1,2)^.

Performed for more than forty years, phacoemulsification uses ultrasound to break and emulsify the nucleus, allowing smaller incisions, and consequently earlier visual rehabilitation and lower complication rates compared to previous techniques^(3)^. It is currently considered the gold standard for cataract surgery, and despite having low complication rates, surgical success is directly proportional to the surgeon’s experience^(4)^.

In 2001, femtosecond laser technology was first introduced for creating the corneal flap in refractive surgeries^(5)^. Later, its use was extended to cataract surgery or femtosecond laser assisted cataract surgery (FLACS), which is a partially automated procedure, and helps conduct safer, faster, and more accurate surgeries^(6)^. There are several femtosecond laser manufacturers on the market, each with its own characteristics, including LDV Z8 (Ziemer Ophthalmic), Intralaser (Abbot Medical Optics), Visumax (Zeiss-Meditec), Femtec (Technolas Perfect Visuion), FS200 (Alcon -Wavelight), LenSx (Alcon), CataLYS (Optimedica), and Victus (Baush Lomb)^(7)^.

Some benefits of using FLACS include greater precision and predictability in corneal incisions and capsulotomy, making it easier to implant the intraocular lens, and decrease time of ultrasound use during phacoemulsification, causing less corneal cellular damage^(8,9)^. It can also be helpful in more difficult cases, including sublu xated cataracts, white cataracts, shallow anterior chamber. Despite the benefits mentioned, some studies have reported complications associated with the use of femtosecond laser, such as capsular block syndrome with the consequent posterior capsule rupture and miosis after laser application^(10,11)^.

Intraoperative miosis hinders nuclear fragmentation, cortex removal, and intraocular implantation of the lens, in addition to increasing the risk of posterior capsule rupture^(12)^. Thus, intraoperative miosis can reverse the benefits gained from laser application. The main purpose of this study is to evaluate and compare variations in pupillary diameter before and after cataract surgery by conventional phacoemulsification versus FLACS, using the LDV Z8 (Ziemer Ophthalmic). To date, no study has reported this laser and intraoperative miosis in the literature. We also evaluated the relationship between pupillary diameter and surgery time and ultrasound time, and variations in pupillary diameter in the intraoperative period.

## METHODS

This is a prospective, observational study approved by the Ethics Committee of the Institution. The research was in accordance to the tenets of the Declaration of Helsinki and informed consent was obtained from all participants after the study had been fully explained. Inclusion criteria were patients with significant nucleus opacity undergoing cataract surgery. Exclusion criteria were coexistent retinal pathology, glaucoma or previous angle-closure glaucoma, subluxated lens or zonular weakness, and pseudoexfoliation syndrome or uveitis. All patients underwent cataract surgery through the Stellaris phacoemulsification platform (Bausch + Lomb, Rochester, New York). The control group included patients submitted to cataract surgery by conventional phacoemulsification, and the study group included patients submitted to FLACS, using the Ziemer LDV Z8 femtosecond laser (Ziemer Ophthalmic Systems AG, Switzerland).

All surgeries were performed under topical anesthesia by the same experienced surgeon (JEG). On the day before surgery, all patients used topical non-steroidal anti-inflammatory drops (0.5% trometamol ketorolac) 3 times a day (8/8 h). Preoperatively, a combination of two mydriatic drops was used three times before surgery: tropicamide and 2.5% phenylephrine. For patients in the study group, corneal incisions (the main incision and an accessory paracentesis), capsulotomy, and fragmentation of the nucleus into six parts were performed using the Ziemer LDV Z8 laser platform, followed immediately for the continuation of their surgeries. The capsulotomy diameter was 4.8 mm, with 105% laser energy. To quantify pupillary size, two horizontal measurements were performed using a surgical compass by the same surgeon who performed the surgeries. In the control group, two measurements of the pupillary diameter were performed: first, before phacoemulsification; the second, at the end of the surgery. In the femtosecond group, three measurements of pupillary diameter were taken: 1) before laser application, 2) after laser application, and 3) at the end of the surgery (phacoemulsification). Use of mydriatics or intraoperative complications was documented. Total surgical duration and phacoemulsification time were also analyzed in both groups.

All data collected were analyzed using the SPSS statistical software (version 16, SPSS, Inc.). For an effect size of 0.8, at a significance level of 0.05, and a sample power of 75%, a sample size of at least 23 eyes per treatment group was required to detect a difference between groups. To analyze changes in pupil size in each group, the paired *t*-test was used. To compare pupil size between the groups at each time, the unpaired *t*-test was used. Differences in pupil size between groups at different times were compared using an analysis of variance (ANOVA). When both eyes were available for analysis, we obtained the mean values between both eyes and used this mean to avoid bias introduced using data with high correlation, like both eyes of the same patient.

## RESULTS

Seventy-nine eyes from 67 patients, including 29 (43%) men and 38 (57%) women, were evaluated. In the control group, 23 (30%) eyes were included, and the mean age was 71.3 ± 7.6 years (range, 58-86). In the femtosecond group, 56 eyes (70%) were included, and the mean age was 68.9 ± 8.6 years (range, 56-88).

Table 1 shows the results for pupil size variation in both groups. There was no significant difference between pupil diameter before surgery in both groups (8.69 ± 0.44 mm in the study group *vs.* 8.63 ± 0.72 mm in the control group; *p*=0.643). In the femtosecond group, there was a transient increase in the pupillary diameter after laser application (and immediately before the start of phacoemulsification), from 8.69 ± 0.44 mm to 9.12 ± 0.56 mm (*p*=0.003).

**Table 1:** Horizontal pupil size diameter, before and after cataract surgery (± SD, in mm).

	Preoperative pupil size	Pupil size after laser and before phaco	Pupil size at the end of surgery	Total pupil variation
Femtosecond group (study)	8.69 ± 0.44	9.12 ± 0.56 (p=0.003^*^ vs preoperative size)	7.96 ± 0.98 (p<0.001^*^ *vs* preoperative size)	1.16 ± 0.88
Control group	8.63 ± 0.72	*Not applicable*	7.78 ± 0.95 (p<0,001^*^ *vs* preoperative size)	0.85 ± 0.68
Significance (Study vs control group)	p=0.643, *ns*		p=0.480, *ns*	p=0.155, *ns*

* = *Statistically significant. ns*= not significant;

*SD*= standard deviation.

We created a new variable, the pupil variation, which analyses the total change in pupil variation, taking into consideration transient increase after femtosecond laser application in the study group. In the control group, a variation of 0.85 ± 0.68 mm (difference between pupil diameter before phaco and after surgery) was observed. In the femto group, a variation of 1.16 ± 0.88 mm (difference between pupil size after femto pre-treatment and after surgery; *p*=0.155) was observed. This indicates a trend towards higher pupil variation in the femto group, which although not statically significant, was close to the limit of significance.

There was no statistically significant difference in mean surgery time and mean ultrasound time between both groups (Table 2).

**Table 2:** Mean time of surgery and ultrasound between groups (± SD)

	Femtosecond group	Control group	p-value
Total surgery duration (minutes)	13.36 ± 1.36 min	13.47 ± 1.65 min	p=0.780, *ns*
Ultrasound time (seconds)	33.36 ± 27.36 s	35.82 ± 24.57 s	p=0.665, *ns*

*ns*= not significant; *SD*= standard deviation.

## DISCUSSION

Femtosecond laser treatment was reported to be associated with pupillary constriction, which may reduce the surgical benefits obtained with the use of this technology^(13)^. The exact mechanism of femto-induced miosis is unknown, but it appears to be triggered by inflammatory mediators^(14)^. Increased levels of prostaglandins (PGs) and interleukins IL-1β and IL-6 were observed in patients submitted to FLACS. High levels of inflammatory mediators have do not correlate with patient age, cataract density, suction time, laser time, or creation of corneal incisions by the laser^(14,15)^.

The use of topical non-steroidal anti-inflammatory drugs (NSAIDs) in the preoperative period has shown reduced intraoperative miosis risk during the procedure; however, the time of application was undetermined^(13)^. In our routine, all patients received a drop of NSAIDs three times a day before surgery as preoperative preparation.

In our study, significant miosis at the end of surgery occurred in both groups, varying from 8.69 ± 0.44 mm to 7.96 ± 0.98 mm in the study group (*p*<0.001) and from 8.63 ± 0.72 mm to 7.78 ± 0.95 mm in the control group, (*p*<0.001). No significant difference was found in pupil size at the end of surgery between the groups (*p*=0.480). These results suggest that there is a similar decrease in pupillary diameter size during cataract surgery in both procedures.

However, we can affirm that miosis was more intense in the group submitted to femtosecond laser, because the pupil first increased from 8.69 ± 0.44 mm to 9.12 ± 0.56 mm (p=0.003) after laser and then decreased to 7.96 ± 0.98 mm after phacoemulsification, with a total variation of 1.16 ± 0.88 mm vs. a variation of 0.85 ± 0.68 mm (*p*=0.155) in the control group. It indicates a trend towards higher variation in the study group, however close to the limit of significance in statistical test. Such finding is not yet described in the literature, however some theories may explain this phenomenon, such as the suction energy (vacuum), which is applied during the execution of the laser and leads to transient mydriasis or the longer exposure time to mydriatic eye drops instilled before surgery.

Although Femto LDV Z8 was reported to use a low energy system, which generates few bubbles during lens fragmentation, it triggered the release of more prostaglandins^(16)^ and the use of NSAIDs may help prevent miosis induced by the laser. Although it may have attenuated it, we wondered if other factors contributed to miosis, such as smaller pupil size in the preoperative period and higher ultrasound time, which generates free radicals, in surgery by conventional phacoemulsification. Mean surgery time and mean ultrasound time in both groups were similar (13.36 ± 1.36 min vs. 13.47 ± 1.65 min, *p*=0.780) and (33.36 ± 27.36 s vs. 35.82 ± 24.57 s, *p*=0.665), respectively, with no difference in the level of free radicals, which could explain the similarity of pupillary constriction between the two procedures.

There are some limitations to our study. First, the degree of opacity of the lens nucleus and manipulation during the procedure were not considered, which may influence surgery time and ultrasound time. However, as there were no differences in these parameters between both groups and laser energy used was the same in all cases, we believe that cataract density did not influence the results. Second, we did not evaluate other factors that could influence pupillary diameter, such as the presence of diabetes, anterior chamber depth, and axial length.

Studies have shown that FLACS has several advantages over conventional phaco, such as a higher precision capsulotomy and better intraocular lens positioning, leading to superior refractive results^(17)^. However, our study confirmed that FLACS may present disadvantages such as the occurrence of intraoperative miosis. Although miosis may occur in conventional phaco and the average pupil size was similar in both groups at the end of the surgery, it was more pronounced in the femto group, as demonstrated by higher pupil variation in this group.

Therefore, when choosing a technique for cataract surgery, the surgeon must observe the preoperative pupil size and consider that femtosecond use may lead to intraoperative miosis, which would nullify the benefits acquired with the application of the laser. Further studies are needed to evaluate from which pupillary diameter size it would be more advantageous choosing the femtosecond laser method over conventional phaco to combine its refractive advantages with less intraoperative complications.
